# Longitudinal changes in complete avascular area assessed using anterior segmental optical coherence tomography angiography in filtering trabeculectomy bleb

**DOI:** 10.1038/s41598-021-02871-2

**Published:** 2021-12-03

**Authors:** Ai Kido, Tadamichi Akagi, Hanako Ohashi Ikeda, Takanori Kameda, Kenji Suda, Masahiro Miyake, Tomoko Hasegawa, Shogo Numa, Akitaka Tsujikawa

**Affiliations:** 1 Department of Ophthalmology and Visual Sciences, Graduate School of Medicine, Kyoto University, Kyoto, Japan; 2 Division of Ophthalmology and Visual Science, Graduate School of Medical and Dental Sciences, Niigata University, 1-757, Asahimachi-dori, Chuo-ku, Niigata, 951-8510 Japan

**Keywords:** Medical research, Biomarkers

## Abstract

Optical coherence tomography angiography (OCTA) is a new technique for non-invasive imaging of blood vessels, allowing combined evaluation of both deep and surface vessels. The purpose of this study was to evaluate the post-trabeculectomy longitudinal changes in complete avascular area (CAA) of filtering blebs using anterior segment (AS-) OCTA and their association with surgical outcomes. This study included 57 eyes of 53 patients who had undergone trabeculectomy with mitomycin C. AS-OCTA images of filtering bleb were acquired at 3 and 6 months after trabeculectomy, and at 1 month in possible cases. CAAs, regions where complete blood flow was not depicted in AS-OCTA images, were evaluated for their presence, extent, and change over time. CAAs were detected in 37 eyes (65%) and 33 eyes (58%) at 3 and 6 months postoperatively, respectively. The extent of CAAs reduced over time after surgery in most cases. No parameters related to CAAs were significantly associated with surgical success (i.e., intraocular pressure (IOP) ≤ 12 mmHg and IOP reduction > 20% without medication). In conclusion, although it is difficult to predict surgical success by CAA itself, AS-OCTA may be useful for the objective evaluation of the vascularity of filtering blebs.

## Introduction

Glaucoma is one of the leading causes of blindness worldwide^1, 2^. Lowering intraocular pressure (IOP) is the only established treatment for delaying the progression of irreversible glaucomatous visual field loss^3–8^. Trabeculectomy is the gold standard in glaucoma surgery^9–11^. Anterior segment (AS-) optical coherence tomography (OCT) has enabled to evaluate the morphology of trabeculectomy filtering bleb, such as bleb wall thickness, internal cavity, and microcysts, which are well known to be associated with surgical success^12, 13^. Vascularity of the filtering bleb is an important factor associated with the surgical outcome of slit-lamp microscopy^14–16^. However, AS-OCT has several limitations for the evaluation of vascularity of filtering bleb: it is highly influenced by the background light, is difficult in the deeper vasculature, and has less objectivity^17, 18^.

OCT angiography (OCTA) is a new technique for non-invasive imaging of blood vessels. It can obtain signals at any depth of tissue, allowing combined evaluation of both deep and surface vessels^19, 20^. While OCTA is commonly used to evaluate blood flow in the retina and choroid^21–25^, AS-OCTA also can visualize blood flow in the iris, conjunctiva, and sclera^26–30^. We previously reported that AS-OCTA is useful for detecting the avascular area in the filtering trabeculectomy bleb^28^. However, the longitudinal changes in AS-OCTA avascular areas and their associations with surgical outcomes have been unknown.

The purpose of this study was to investigate the post-trabeculectomy longitudinal changes in avascular area in filtering blebs using AS-OCTA and their association with surgical outcomes.

## Results

### Characteristics of the target population

In total, 74 of 69 consecutive patients were enrolled in the study. Of these, 14 eyes were excluded because of history of intraocular surgery and 3 eyes were excluded due to lack of 6 months follow-up. Finally, 57 eyes from 53 patients were analyzed. The baseline characteristics of the total participants are summarized in Table 1. The mean patient age was 65.2 ± 13.2 years. There were 35 eyes diagnosed with primary open angle glaucoma (POAG); 3 eyes, primary angle closure glaucoma; and 19 eyes, secondary glaucoma (8 exfoliation glaucoma, 9 uveitic glaucoma, and 2 neovascular glaucoma). The mean preoperative IOP was 22.1 ± 8.4 mm Hg and the mean postoperative IOP at 12 months was 11.1 ± 2.9 mm Hg. Antiglaucoma eye drops were needed in 9 eyes, and needling procedure was performed in 5 eyes in up to 12 months postoperatively.

**Table 1:** Patient characteristics.

Variables	Total	Successful	Unsuccessful	P-value
	N = 57	N = 36	N = 21	
Age (years)	65.2 ± 13.2	66.6 ± 11.6	62.9 ± 15.5	0.57*
Sex (male/female)	34/23	23/13	11/10	0.27**
Spherical equivalents (diopters)	− 3.32 ± 3.77	− 2.76 ± 3.46	− 4.30 ± 4.16	0.16*
Axial length (mm)	25.12 ± 1.73	24.96 ± 1.73	25.40 ± 1.75	0.27*
Central corneal thickness (μm)	524.5 ± 38.2	513.7 ± 28.1	543.3 ± 46.5	0.01*
Visual field, mean deviation (dB)	− 17.67 ± 6.18	− 17.81 ± 5.83	− 17.44 ± 6.87	0.89*
Preoperative IOP (mmHg)	22.1 ± 8.4	21.94 ± 7.56	22.33 ± 9.86	0.78*
Preoperative eyedrops score	3.9 ± 1.0	4.1 ± 0.9	3.7 ± 1.1	0.26*
Diagnosis (POAG/PACG, secondary)	38/19	24/12	14/7	0.99**
Operation method (TLE one/ TLE combined with cataract surgery)	45/12	28/8	17/4	0.99**
Postoperative IOP (mmHg)	11.1 ± 2.9	9.6 ± 2.1	13.6 ± 2.4	< 0.001*
**Additional treatment**				
Topical anti-glaucoma medication	16% (9/57)	0% (0/36)	43% (9/21)	< 0.001**
Needling	9% (5/57)	6% (2/36)	14% (3/21)	0.35**
**Presence of CAA in bleb**				
1 month after TLE	78% (28/36)	74% (17/23)	85% (11/13)	0.68**
3 months after TLE	65% (37/57)	67% (24/36)	62% (13/21)	0.78**
6 months after TLE	58% (33/57)	58% (21/36)	57% (12/21)	0.99**
**Extent of CAA (%)**^**a**^				
1 month after TLE	15.58 ± 12.22	14.38 ± 11.29	17.70 ± 13.95	0.62*
3 months after TLE	10.10 ± 10.71	11.31 ± 11.33	8.01 ± 9.44	0.34*
6 months after TLE	8.23 ± 11.32	9.83 ± 13.26	5.51 ± 6.23	0.29*
Change of CAA (%)^b^	− 1.87 ± 7.47	− 1.49 ± 8.06	− 2.50 ± 6.46	0.93*

*CAA* complete avascular area, *IOP* intraocular pressure, *POAG* primary open-angle glaucoma, *PACG* primary angle closure glaucoma, *TLE* trabeculectomy.

The following statistical tests are conducted to examine differences between the successful and unsuccessful groups: *Wilcoxon rank sum test; **Chi-square test.

^a^The complete avascular area is measured in pixels, divided by 1,048,576 (1024 × 1024) pixels which is the entire imaging area, and converted to percentage.

^b^The change in complete avascular area is calculated by subtracting the extent of the complete avascular area after 3 months from that after 6 months.

### Longitudinal changes in complete avascular area in bleb assessed using AS-OCTA

**Figure 1:**
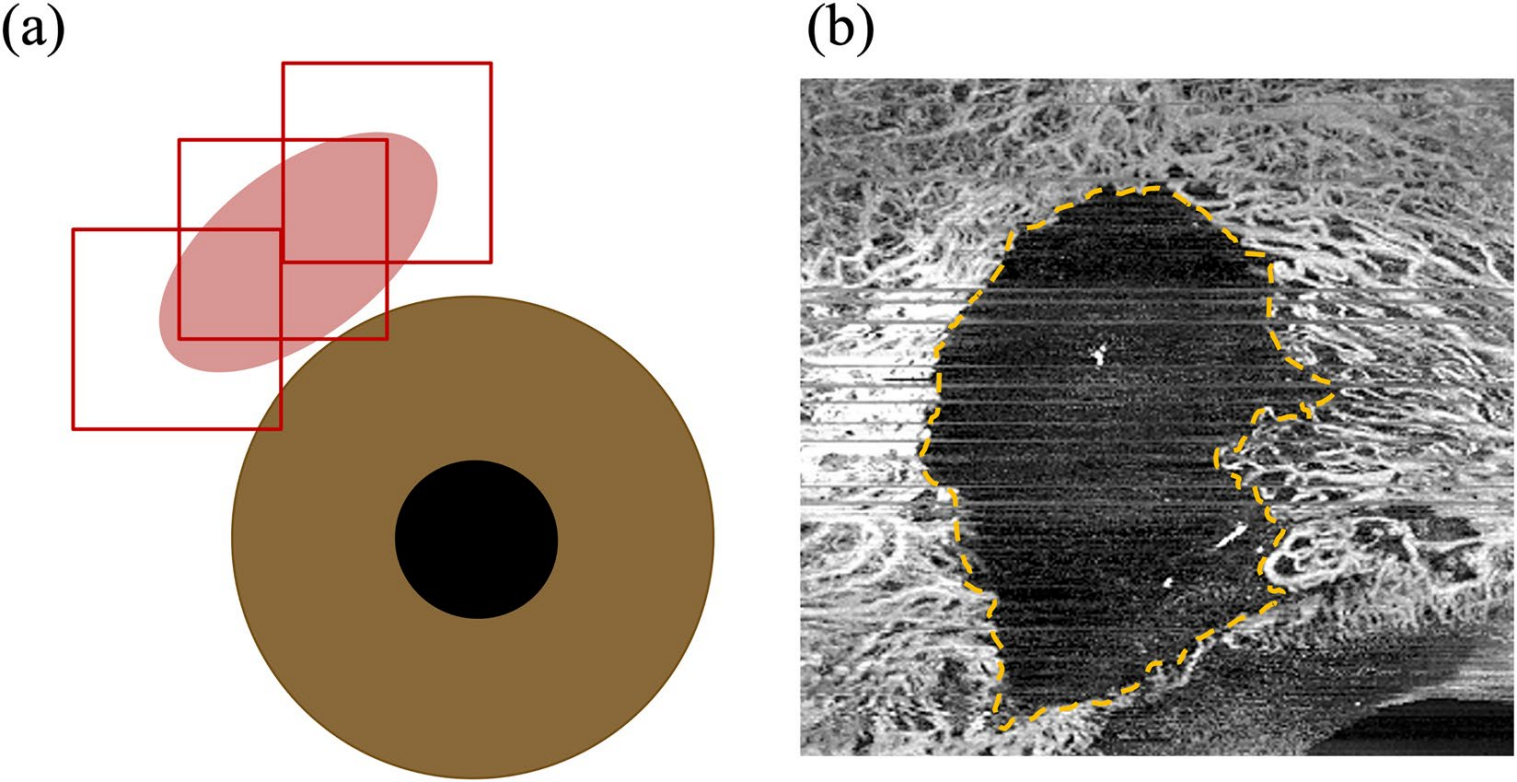
Anterior segment optical coherence tomography angiography assessment of complete avascular area (CAA) in the filtering bleb. (**a**) 3 × 3 mm AS-OCTA images are taken at the center of the filtering bleb, with the scleral flap and its nasal and temporal sides scanned in the same manner. (**b**) The extent of CAA (%) is calculated by dividing CAA in pixels by the total number of pixels (1,048,576 = 1024 × 1024 pixels).

**Figure 2:**
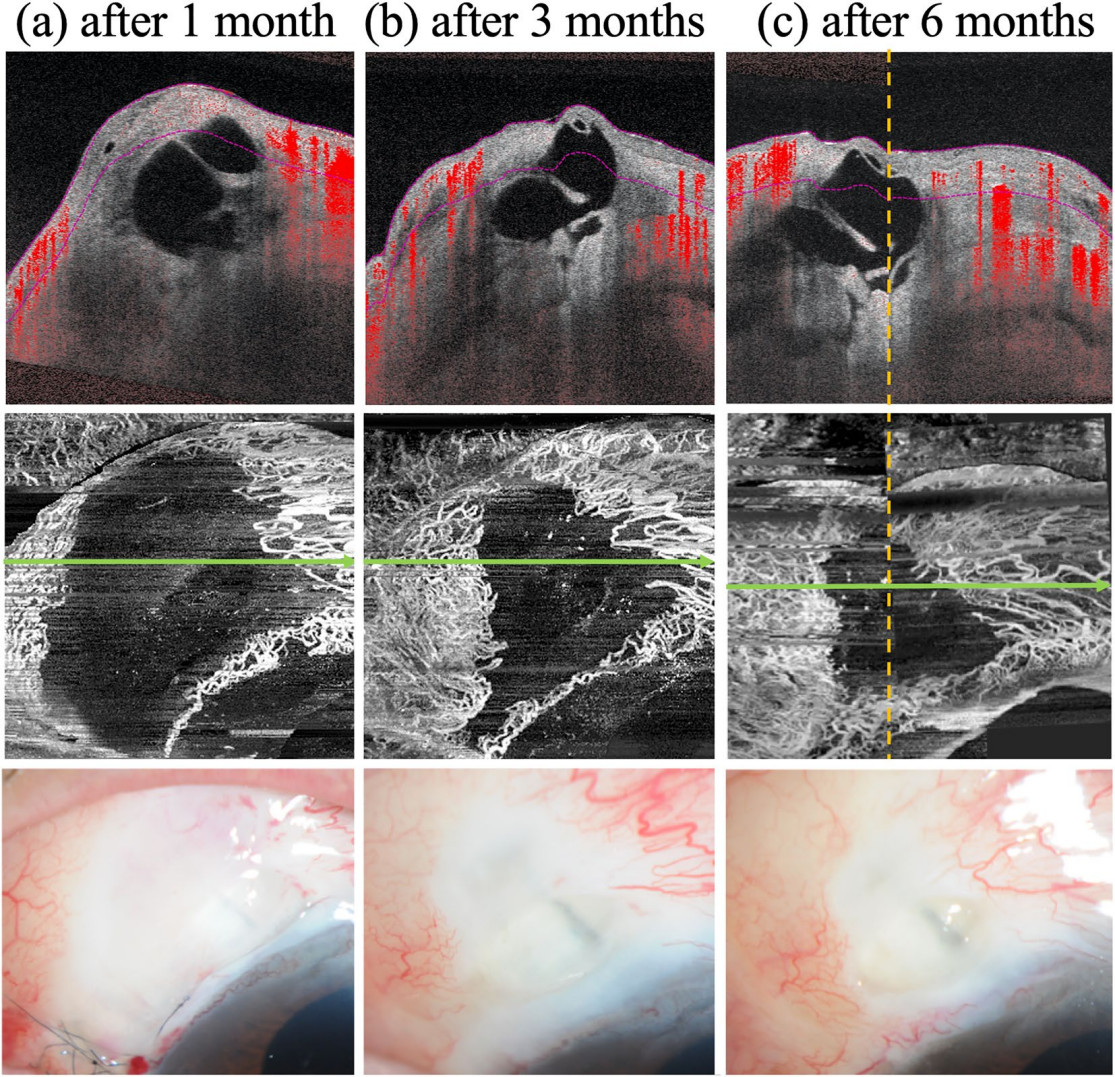
A typical case in which the complete avascular area (CAA) is reduced over time. Cross-sectional OCTA images overlying the B-scan images (upper), AS-OCTA en face images (middle), and slit-lamp microscopy photographs (bottom) at 1 (**a**), 3 (**b**), and 6 (**c**) months postoperatively. CAAs detected in the AS-OCTA are identical to the avascular area observed in the slit-lamp microscopy. The CAA decreases in size postoperatively.

**Figure 3:**
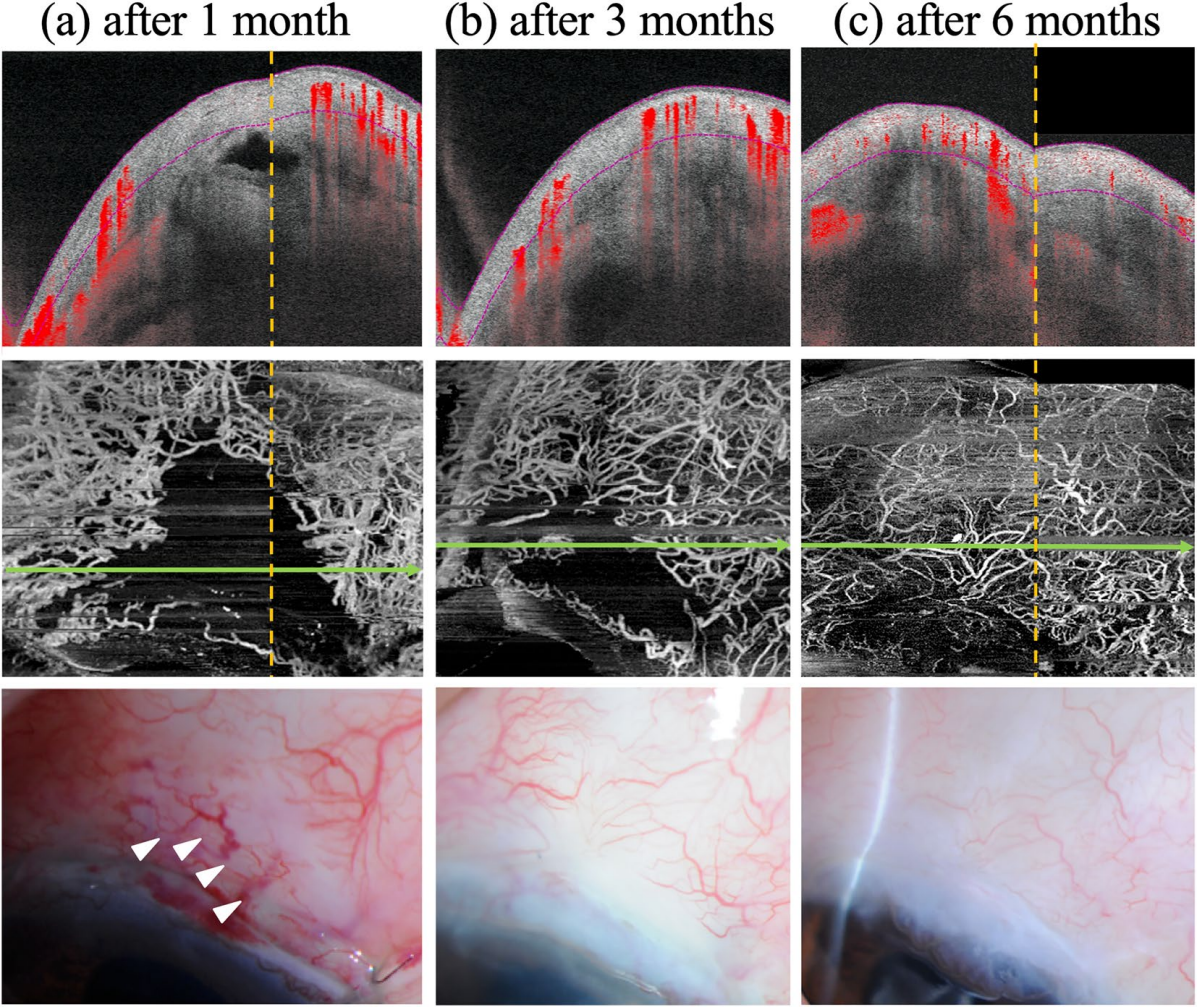
A case with discrepancy between AS-OCTA and slit-lamp microscopy findings. Cross-sectional OCTA images overlying the B-scan images (upper), AS-OCTA en face images (middle), and slit-lamp microscopy photographs (bottom) at 1 (**a**), 3 (**b**), and 6 (**c**) months postoperatively. The complete avascular area (CAA) decreases as time passes. A marked CAA is observed in the AS-OCTA image at 1 month after surgery (**a**, middle), but meandering blood vessel-like structures exist in the slit-lamp microscopy photograph (**a**, bottom, white arrowheads).

Complete avascular areas (CAAs) in the filtering bleb were detected in the AS-OCTA images in 37 eyes (65%) and 33 eyes (58%) at 3 and 6 months postoperatively, respectively (Fig. 1 and Table 1). OCTA images could be analyzed 1 month postoperatively in 36 eyes, and CAAs were observed in 28 eyes (78%). Figure 2 shows a typical case in which the CAA was reduced over time. The CAA detected in the AS-OCTA was identical to the avascular area that observed in the slit-lamp microscopy (Figs. 2, 3B,C) in most cases, but not in some cases (Fig. 3A).

### Comparison between the successful and unsuccessful surgery groups

At 12 months postoperatively, surgery was successful and unsuccessful in 36 and 21 eyes, respectively. There were no statistically significant differences in the background factors, including preoperative IOP, diagnosis, and combination with cataract surgery, between the two groups (Table 1). The presence and extent of the CAA were also not significantly different between the groups at any observation period (Table 1). The longitudinal change of the extent was smaller in the successful group than in the unsuccessful group (− 1.49 ± 8.06 vs − 2.50 ± 6.46), but the deviation was so large that the difference was not statistically significant (P = 0.93; Table 1).

### Relationship between surgical success and complete avascular area assessed using OCTA

Table 2 shows the association between the surgical success and the parameters of CAA. The presence of CAA at any time point was not associated with the surgical success in the unadjusted and adjusted analyses. The extent or longitudinal change of CAAs was also not significantly associated with the surgical success. The power of the current study was greater than 0.8 for effect sizes higher than a Cohen’s value 0.37.

**Table 2:** Association between surgical success and complete avascular area (CAA).

Variables	Unadjusted Risk Ratio (95% CI)	P-value	Adjusted Risk Ratio (95% CI)	P-value
**Presence of CAA**				
1 month after TLE	0.52 (0.07–2.72)	0.46	0.37 (0.04–2.45)	0.33*
3 months after TLE	1.23 (0.39–3.78)	0.72	1.10 (0.33–3.57)	0.88*
6 months after TLE	1.05 (0.35–3.12)	0.93	0.95 (0.29–2.99)	0.93*
**Extent of CAA**				
1 month after TLE	0.98 (0.92–1.03)	0.43	0.97 (0.91–1.04)	0.40*
3 months after TLE	1.03 (0.98–1.09)	0.26	1.03 (0.97–1.09)	0.32*
6 months after TLE	1.06 (0.99–1.13)	0.17	1.05 (0.99–1.14)	0.21*
Change of CAA	1.02 (0.95–1.10)	0.62	1.02 (0.94–1.10)	0.64*

*Multivariable analysis is performed with surgical success as the objective variable, in which each of the items related to CAA (presence, extent, and change) is adjusted for age, operation method, and preoperative IOP.

## Discussion

This study showed that AS-OCTA could be used for longitudinal assessments of CAAs of the filtering blebs after trabeculectomy surgery. The CAA was frequently observed in the early postoperative period and had a tendency to become smaller as time passed. However, the presence, extent, or longitudinal change of CAAs was not significantly associated with the surgical success. To our best knowledge, this is the first study to focus on the CAA assessed using AS-OCTA.

The Indiana Bleb Appearance Grading Scale and Moorfields Bleb Grading System are well-known bleb grading systems^14–16^. In these grading systems, bleb vascularity is evaluated using standard photographs, which are subjective and difficult to apply in quantification analyses. In the current study, the CAA assessed by AS-OCTA was quite identical to the avascular area observed in slit-lamp photograph in most cases, indicating that AS-OCTA can be used alternatively to assess the bleb avascular area. The results of the association between the existence of CAA and the Indiana Bleb Appearance Grading Scale assessment at 6 months post-trabeculectomy in each case are summarized in Supplementary Table 1. Most of the filtering blebs with CAAs were classified as V0 (avascular white) or V1 (avascular cystic), which indicates that CAAs assessed by AS-OCTA may be closely linked to the vascularity in the conventional bleb grading system.

There were obvious differences between the AS-OCTA CAA and slit-lamp avascular area in some cases. In these cases, some vessels observed in the slit-lamp images were not detected in the AS-OCTA images (Fig. 3A). Although it is unclear whether blood flow was absent or too slow to detect using AS-OCTA in these AS-OCTA negative blood vessels, we speculate that conjunctival surgical incision may disrupt the blood vessels, subsequently leading to reconstructed or reperfused bleb vasculature. This speculation may explain our results that the CAA reduced over time after surgery. In this study, trabeculectomy was performed using fornix-based conjunctival flap with radial conjunctival incision. Further studies including other types of filtering surgery, such as limbal-based trabeculectomy or filtering surgery without conjunctival incision, might reveal the influences of conjunctival incision on the process of bleb CAA formation in more detail.

The present study revealed that the CAA shrank over time after surgery. One of the possible reasons for this, as mentioned in the previous paragraph, is that the blood flow disrupted intraoperatively may be reconstructed or reperfused postoperatively. Another possible reason is the influence of mitomycin C, which is used during surgery to prevent postoperative scarring of the bleb. The longitudinal reduction in the effect of mitomycin C might contribute to the reduction of CAA. Another possible cause is the involvement of the fluid pressure in the filtering blebs. In almost all our cases with large cysts in the filtering blebs, CAAs existed in the area with cysts and scleral flap. The flow pressure of filtered aqueous humor may affect CAA.

In the present study, there was no significant association between the longitudinal changes of CAA and surgical success. Our criteria for surgical success might be stringent because the target IOP after trabeculectomy is usually very low in our clinical setting. To confirm whether our results are dependent on the success criteria, we applied different success criteria (IOP ≤ 14 mm Hg or IOP ≤ 16 mm Hg), and similar results were obtained. AS-OCTA CAAs were not significantly associated with surgical success in the different success criteria (Supplementary Table 2). We also included various types of glaucoma aside from POAG, and the result was similar, even when we included only 35 POAG eyes in the analysis (data not shown). The results of power calculation showed that, if the true effect size was higher than a Cohen’s value of 0.37, the lack of statistical significance suggests no true difference.

Postoperative vascularization and scarring of the filtering blebs are known to interfere with the filtration capacity of well-functioning blebs, resulting in increased IOP^31, 32^. As such, we speculated that a strong vascular growth, i.e., a small extent of the CAA or a decrease of CAA, might be negatively associated with surgical success. A previous study investigating trabeculectomy bleb using AS-OCTA showed that the difference in vessel area between 1 week and 1 month postoperatively may predict IOP at 6 months postoperatively^33^. Moreover, the surgical success of trabeculectomy is known to be closely associated with the bleb morphology, such as bleb wall thickness, internal cavity, microcysts, subflap space, and internal ostium^12, 13, 17, 34^. The surgical outcomes of trabeculectomy may be affected by a complex interaction of various factors, and our results indicate that AS-OCTA CAA alone is probably inadequate to predict surgical success. However, because the bleb vascularity, which can be precisely and quantitatively assessed by AS-OCTA, is closely associated with the scarring process of bleb, further studies are needed to establish useful methods for better assessments of bleb.

The present study has the following limitations. First, the current OCTA has been developed for the posterior segment of the eye. When acquiring OCTA images of the retina, an eye-tracking system is effective for imaging the same area longitudinally. However, when acquiring OCTA images of the anterior segment of the eye, it is difficult to image the exact same area using the current OCTA system. We addressed this limitation by using the scleral flap as a mark to adjust the location. The improvement of the AS-OCTA system including software would be helpful for further investigations. Second, although AS-OCTA images were successfully acquired, they still had too much noise. It is known that averaging multiple images helps improve the image quality of posterior segment OCTA^35^. In the future, averaging AS-OCTA images may help to improve image quality.

## Conclusion

The CAA could be visualized longitudinally and had a tendency to reduce or disappear with time after trabeculectomy surgery. AS-OCTA may be useful for objective evaluation of the vascularity of the filtering blebs.

## Methods

### Study design

This prospective consecutive case series study was conducted from two ongoing prospective studies at Kyoto University Hospital: the Kyoto University Glaucoma Progression Study (registered with the University Hospital Medical Information Network [UMIN] Clinical Trial Registry of Japan [UMIN000019854]) and the Clarification of Eye Diseases using OCTA (UMIN000028853). Both study protocols were approved by the Institutional Review Board and Ethics Committee of Kyoto University Graduate School and Faculty of Medicine (No. R0625 and R0656). All investigations in the current study adhered to the principles of the Declaration of Helsinki. Written informed consent was obtained from the patients after the study design and risks and benefits of participation were fully explained. Consent to publish was also confirmed for cases whose data was used in the current study.

### Participants

The study subjects were glaucoma patients who underwent trabeculectomy with mitomycin C at Kyoto University Hospital between January 1, 2018 and April 30, 2019 and agreed to participate in this study. The inclusion criterion was a negative history of intraocular surgery (excluding cataract surgery and trabecular-targeted MIGS without conjunctival incision).

### Surgical technique

**Figure 4:**
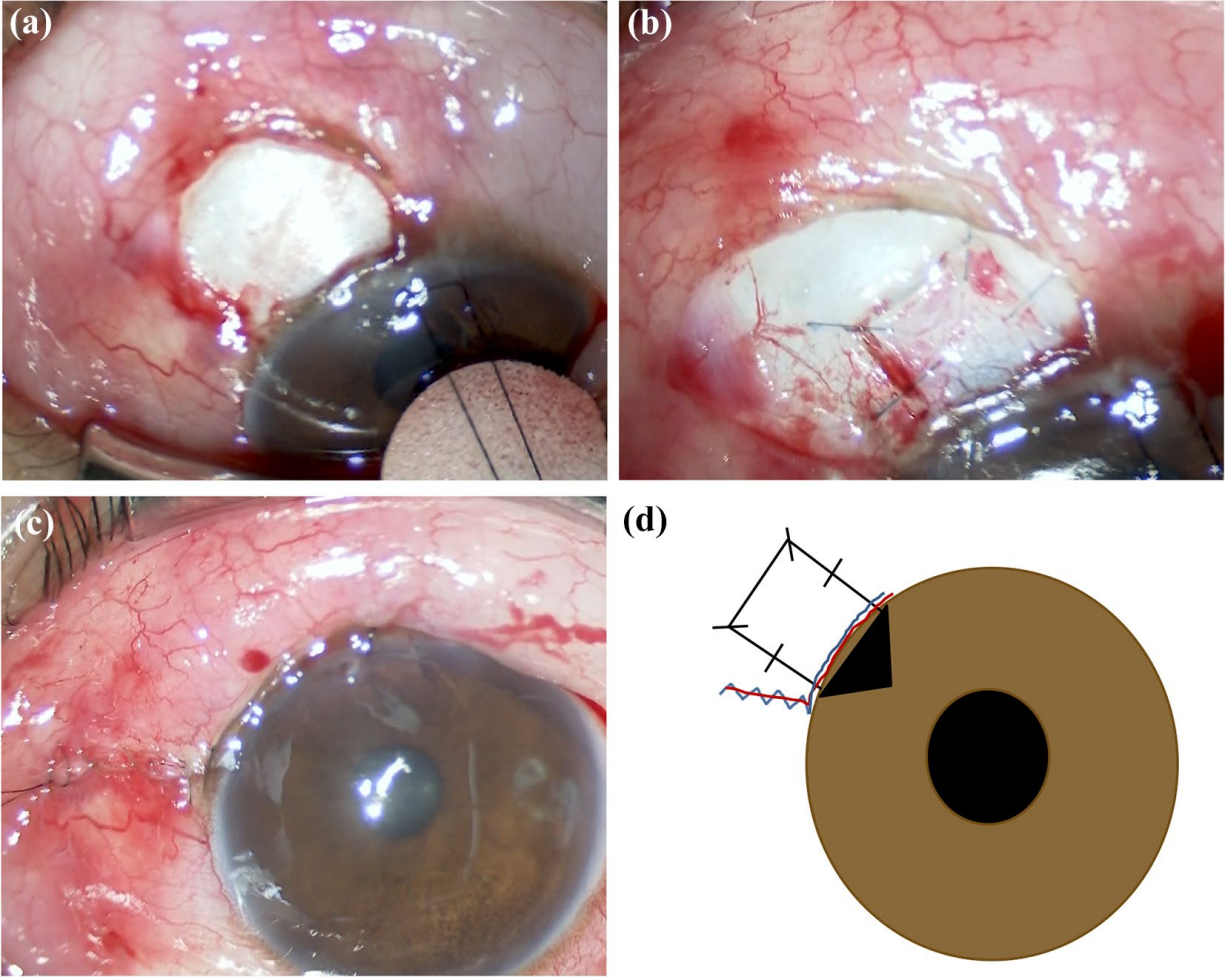
Procedure of trabeculectomy. Intraoperative images of trabeculectomy in this study (**a**–**c**) and a schema of the incision sites (**d**). A fornix-based conjunctival flap with small radial incision (**a**), a 3 × 3 mm half-layer square scleral flap formation (**b**), and suturing of the scleral flap and conjunctiva using 10–0 nylon (**c**) are performed. (**d**) Schema of the operative field.

In this study, trabeculectomy was performed with a fornix-based conjunctival flap and a 3 × 3 mm half-layer square scleral flap (Fig. 4). A sponge soaked with mitomycin C (0.4 mg/mL, Kyowa Hakko Kirin, Tokyo, Japan) was applied for 3–4 minutes to the posterior surface of the conjunctiva, Tenon capsule, the adjacent episcleral tissue, and the scleral flap. The block of trabecular meshwork tissue was excised, and peripheral iridectomy was performed. The scleral flap was closed with four to five 10–0 nylon sutures, and the conjunctiva was closed using 10–0 nylon sutures.

The treated eyes were prescribed topical steroid for 3 months (unpreserved betamethasone 0.1% eye drops four times a day) and a topical antibiotic for the first month (moxifloxacin 0.5% eye-drops four times daily). Laser suture lysis was performed within 1 month postoperatively when the postoperative IOP was higher than the target IOP. Needle revision was performed for cases of unsuccessful laser suture lysis. Glaucoma medication was resumed when the postoperative IOP exceeded the target IOP after laser suture lysis and/or needle revision.

### Criteria for surgical success

Surgical success was defined as an IOP ≤ 12 mmHg and a reduction of more than 20% in IOP without medication or additional glaucoma surgeries (excluding needle revision) at 12 months post-trabeculectomy. The subjects were classified into two groups according to the surgical success criteria at 12 months postoperatively: successful or unsuccessful.

### AS-OCTA examination

AS-OCTA examination was performed using a swept-source OCT system (PLEX Elite 9000; Carl Zeiss Meditec, Dublin, California, USA). This instrument has a central wavelength of 1040–1060 nm, a bandwidth of 100 nm, an A-scan depth of 3.0 mm in tissue, and a full-width at half-maximum axial resolution of approximately 5 μm in tissue. The instrument performs 100,000 A-scans per second. AS-OCTA images were acquired using the 10-diopter optical adaptor lens developed by Carl Zeiss Meditec^26, 27^.

### AS-OCTA image acquisition and processing

Postoperative follow-up using AS-OCTA was performed at 3 and 6 months post-trabeculectomy. In addition, AS-OCTA images were obtained at 1 month postoperatively in possible cases. A 3 × 3-mm scan pattern, which consisted of 300 A-scans per B-scan repeated 4 times at each of the 300 B-scan positions, was used to acquire AS-OCTA images as previously reported^27^. The size of this 3 × 3-mm scan pattern corresponds to typical “Retina” dimensions and approximately 6 × 6-mm in AS-OCTA images. For each patient, at least three 3 × 3 mm images were taken at the center of the filtering bleb with the scleral flap and its nasal and temporal sides.

En face images were generated using a built-in soft- ware (Ver. 1.6.0.21130; Carl Zeiss Meditec). Flattening was performed at the level of the conjunctival epithelium, which was misidentified as the inner limiting membrane by the software. The OCTA flow images were developed with an en face maximum projection from the conjunctival epithelium to a depth of 400 μm.

### Evaluation of bleb using AS-OCTA images

Using OCTA images at 1 month, 3 months, and 6 months after trabeculectomy, the complete avascular area was measured in pixels (Fig. 1). The extent was calculated by dividing the pixel of the CAA by 1,048,576 (1024 × 1024) pixels, which is the entire imaging area, and converted to percentage. The change of CAA was calculated by subtracting the extent of the CAA after 3 months from that after 6 months.

### Statistical analysis

All values were presented as the mean ± standard deviation, where applicable. The Wilcoxon signed-rank test was used to compare the mean values between the two groups classified for surgical success, and the Chi-square test and Fisher’s exact probability test were used to compare the proportions. To examine the relationship between AS-OCTA parameters and surgical success, univariate and multivariate analyses with logistic regression models were performed. Multivariate analysis was adjusted for age, operation method, and preoperative IOP. Statistical power was calculated from the actual sample size in this study, assuming high to low effect sizes (Cohen's value 0.5–0.1). All statistical analyses were performed using R software, version 3.2.1 (R Core Team, Vienna, Austria)^36^. P values less than 0.05 were considered statistically significant.

## Supplementary Information

Supplementary Information.

## Data Availability

The raw data of the current study are available at http://www.nature.com/srep, and the program codes are available from the corresponding author on reasonable request.
