# Supplementary material for: Longitudinal changes in complete avascular area assessed using anterior segmental optical coherence tomography angiography in filtering trabeculectomy bleb
Source: Sci Rep. 2021 Dec 3;11:23418. doi: 10.1038/s41598-021-02871-2 (PMC8642474; doi:10.1038/s41598-021-02871-2)
Supplement: Supplementary file 1 — Supplementary Information. [file 41598_2021_2871_MOESM1_ESM.docx]

| Supplementary Table 1. The Indiana Bleb Appearance Grading Scales at 6months post-trabeculectomy of the group with and without complete avascular area (CAA) | | |
| --- | --- | --- |
| Indiana Bleb Appearance Grading Scales | With CAA  N = 33 | Without CAA  N = 24 |
| Bleb Height |  |  |
| H0 (flat bleb) | 2 | 1 |
| H1 (low bleb) | 14 | 13 |
| H2 (medium bleb) | 16 | 10 |
| H3 (high bleb) | 1 | 0 |
| Horizontal Extent |  |  |
| E0 (0 <1 clock hours) | 1 | 0 |
| E1 (1-2 clock hours) | 16 | 13 |
| E2 (>2 - <4 clock hours) | 13 | 11 |
| E3 (4 or > clock hours) | 3 | 0 |
| Vascularity |  |  |
| V0 (avascular white) | 15 | 0 |
| V1 (avascular cystic) | 15 | 2 |
| V2 (mild vascularity) | 3 | 11 |
| V3 (moderate vascularity) | 0 | 11 |
| V4 (extensive vascularity) | 0 | 0 |
| Seidel Test |  |  |
| S0 (no leak) | 33 | 24 |
| S1 (multiple pinpoint leaks) | 0 | 0 |
| S2 (streaming leak) | 0 | 0 |

| Supplementary Table 2. Association between surgical success and complete avascular area (CAA) | | | | |
| --- | --- | --- | --- | --- |
| Variables | Unadjusted RR (95% CI) | P-value | Adjusted RR (95% CI) | P-value |
| Model 1 | | | | |
| Presence of CAA in bleb |  |  |  |  |
| 1 month after TLE | 0.83 (0.11-4.58) | 0.84 | 0.54 (0.06-3.42) | 0.54 * |
| 3 months after TLE | 1.68 (0.50-5.53) | 0.39 | 1.09 (0.28-3.95) | 0.90 * |
| 6 months after TLE | 1.56 (0.48-5.09) | 0.45 | 1.08 (0.29-3.85) | 0.90 * |
| Extent of CAA |  |  |  |  |
| 1 month after TLE | 0.99 (0.93-1.06) | 0.83 | 0.99 (0.93-1.06) | 0.75 * |
| 3 months after TLE | 1.04 (0.98-1.11) | 0.27 | 1.02 (0.96-1.09) | 0.53 * |
| 6 months after TLE | 1.05 (0.98-1.14) | 0.23 | 1.03 (0.96-1.12) | 0.51 * |
| Change of CAA | 1.01 (0.93-1.10) | 0.81 | 1.07 (0.99-1.18) | 0.99 * |
| Model 2 | | | | |
| Presence of CAA in bleb |  |  |  |  |
| 1 month after TLE | 1.22 (0.15-7.10) | 0.83 | 0.72 (0.08-5.04) | 0.75 * |
| 3 months after TLE | 2.31 (0.67-8.11) | 0.18 | 1.53 (0.38-5.89) | 0.54 * |
| 6 months after TLE | 1.53 (0.45-5.25) | 0.49 | 1.06 (0.27-4.03) | 0.93 * |
| Extent of CAA |  |  |  |  |
| 1 month after TLE | 1.00 (0.94-1.07) | 0.98 | 0.99 (0.92-1.06) | 0.77 * |
| 3 months after TLE | 1.05 (0.99-1.13) | 0.17 | 1.04 (0.97-1.12) | 0.32 * |
| 6 months after TLE | 1.05 (0.98-1.16) | 0.23 | 1.03 (0.96-1.14) | 0.49 * |
| Change of CAA | 0.99 (0.91-1.08) | 0.85 | 0.98 (0.89-1.07) | 0.62 * |

Model 1: Surgical success was defined intraocular pressure (IOP) ≤14 mmHg and IOP reduction >20% without medication or additional glaucoma surgeries at 12 months post-trabeculectomy.

Model 2: Surgical success was defined intraocular pressure (IOP) ≤16 mmHg and IOP reduction >20% without medication or additional glaucoma surgeries at 12 months post-trabeculectomy.

* adjusted for age, operation method, and preoperative IOP.

* Multivariable analysis is performed with surgical success as the objective variable, in which each of the items related to CAA (presence, extent, and change) is adjusted for age, operation method, and preoperative IOP.
